# Adrenal Ganglioneuroma: A Case Report

**DOI:** 10.7759/cureus.27634

**Published:** 2022-08-03

**Authors:** Hayat Aynaou, Houda Salhi, Hanan El Ouahabi

**Affiliations:** 1 Department of Endocrinology, Diabetology, Metabolic Diseases and Nutrition, Hassan II University Hospital, Fes, MAR

**Keywords:** adrenalectomy, histology, ps100, adrenal ganglioneuroma, ganglioneuroma

## Abstract

Adrenal ganglioneuroma (AGN) are sympathetic differentiated tumors that originate from neural crest cells. It is a rare benign tumor in children and young adults. These lesions are usually asymptomatic and tend to be hormonally silent. Their discovery is fortuitous in imaging examinations.

Preoperative diagnosis remains difficult, and the gold standard treatment is adrenalectomy. There is a good prognosis after surgery without recurrence. We herein report a case of adrenal ganglioneuroma in a 40-year-old man who benefited from an abdominal CT scan in the face of a complaint of abdominal discomfort and as part of the extension assessment of his colonic adenocarcinoma. Abdominal CT scan with contrast showed a left retroperitoneal mass of triangular shape within the adrenal lodge of tissue density, containing some calcifications not enhanced after injection of contrast product, measuring 90 x 62 mm in diameter (AP x T) with a relative washout calculated at 30%. Biopsy and histological examination of the mass suggested an adrenal ganglioneuroma.

## Introduction

Ganglioneuroma is a benign tumor arising from the neural crest, Schwannien stroma, and connective tissue of the sympathetic channel. It mainly occurs in the posterior mediastinum or retroperitoneum, but also in the adrenal glands and the neck [1,2]. AGN is rare, representing 21% of all GNs [3]. It usually occurs in children and young adults aged 10 to 40 years old, without sexual predominance [3].

They are asymptomatic or they are associated with compressive local effects [4]. The gold standard treatment is adrenalectomy. There is a good prognosis after surgery without recurrence. We describe a case of a 40-year-old man who benefited from an abdominal CT scan in the face of a complaint of abdominal discomfort and as part of the extension assessment of his colonic adenocarcinoma. The particularity of our case is that the discovery of adrenal ganglioneuroma is made in a neoplastic context. The biopsy of the adrenal mass was performed in order to search for an adrenal metastasis. The objective is to demonstrate that the presence of an adrenal mass, (especially if it is unilateral), in a neoplastic context, is not always synonymous with metastases.

## Case presentation

We report the case of the patient, aged 40, followed in oncology for moderately differentiated and infiltrating adenocarcinoma of the right colon, operated on and benefited from chemotherapy. Admitted to our structure for exploration and management of a unilateral left adrenal mass discovered as part of the assessment of extension and pain in the left flank. The physical examination revealed blood pressure at 110/87 mmHg without orthostatic hypotension, heart rate at 80 bpm, weight at 70 kg, height at 1.87 m, and weight loss of 11 kg over one year.

**Figure 1:**
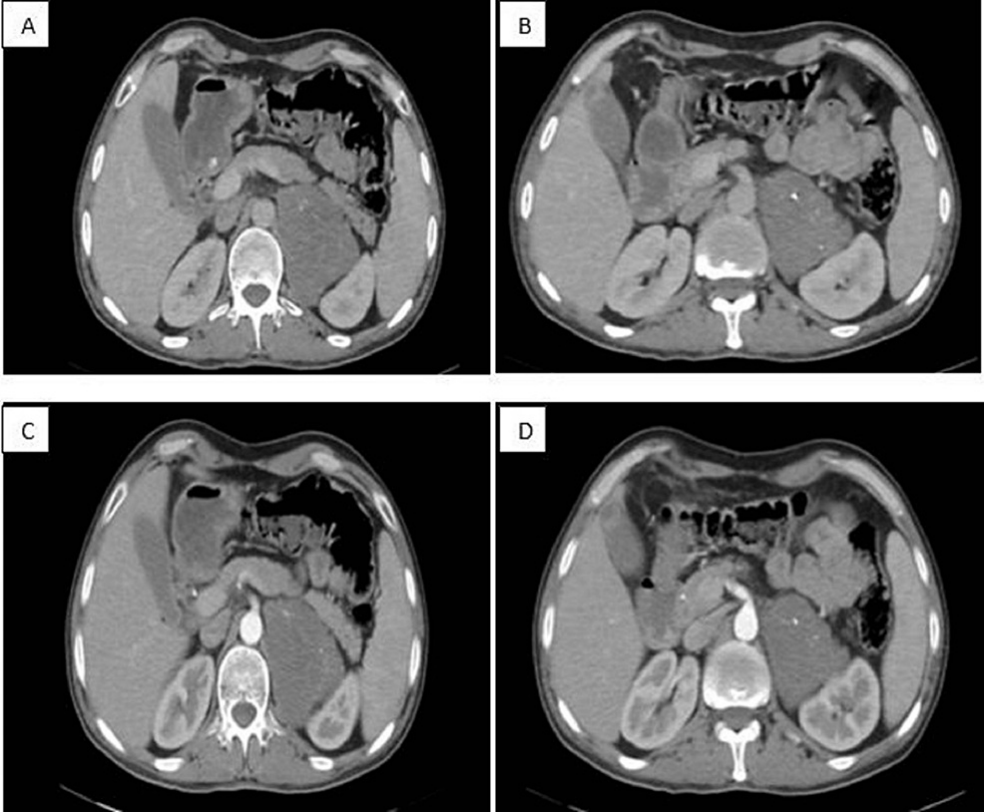
Cross-sectional CT scan C- (A, B), C+ (C,D) showing the triangular left adrenal mass with calcification.

There were no clinical signs of catecholaminergic, mineralocorticoid, or glucocorticoid hypersecretion. Biological assays showed correct serum potassium, negative urinary metanephrines, positive minute braking, and SDHEAs were normal. The abdominal CT scan showed a left retroperitoneal mass of triangular shape within the adrenal lodge of tissue density, containing some calcifications not enhanced after injection of contrast product, measuring 90 x 62 mm in diameter (AP x T) with a relative washout calculated at 30% (Figures 1A-1D).

The patient underwent a biopsy of the mass with an anatomopathological examination that showed a spindle cell tumor, benign in appearance, consisting of a proliferation of regular spindle cells or with rare cytonuclear abnormalities and arranged in elongated or criss-cross bundles. Mitoses are exceptional. Absence of epitheloid and gigantocellular granulomas, absence of necrosis. The complement immunohistochemistry revealed positivity for anti-PS100 antibodies and anti-synaptophysin antibodies in favor of ganglioneuroma. The multidisciplinary decision is not to operate on our patient, given the metastatic character of his colon cancer.

## Discussion

Ganglioneuroma is rare sympathetic differentiated tumors that originate from neural crest cells. The two most common locations are the retroperitoneum (32%-52% of cases) and posterior mediastinum (39%-43%), followed by the cervical region (8%-9%) [5]. Its location in the adrenal gland is unusual and accounts for 21% of all GN [3] and its incidence in adrenalectomized patients ranges from 0.3 to 2.0% [3].

Although GN is generally considered to occur more frequently in young people aged 10 to 40 years old, without sexual predominance [3], some recent studies have shown that it may also be seen between the ages 40 and 50 [6]. AGN are usually asymptomatic (62.9% to 93.3% of cases) [6], and often discovered incidentally as they are slow growing and usually endocrinologically inactive [7]. However, GN can grow large enough to cause symptoms due to mass effect as was demonstrated in our patient.

AGN are generally non-functional but can secrete catecholamines and their metabolites in 37% of cases [8]. These secretions can include vasoactive intestinal peptides [9] and steroid hormones, such as cortisol and testosterone [10].

In our case, the patient presented with nonspecific abdominal pain and had no symptoms of hormonal hypersecretion. The discovery was incidentally on an abdominal scan requested as part of the extension assessment of his colonic adenocarcinoma. Biological assays showed correct serum potassium, negative urinary metanephrines, positive minute braking, and SDHEAs were normal.

On CT scan, the ganglioneuroma appears as a solid, well-circumscribed, and encapsulated mass [11]. The mass appears homogeneous with low attenuation in the pre-contrast phase and appears homogeneous or heterogeneous with increased attenuation in the post-contrast phase [12]. The lesion may also show 42%-60% calcifications which are usually fine and mottled but may be coarse [11,13]. Its size is variable, with an average of 8 cm [14]. In our case, the abdominal CT scan showed a left retroperitoneal mass of triangular shape within the adrenal lodge of tissue density, containing some calcifications not enhanced after injection of contrast product, measuring 90 x 62 mm in diameter (AP x T) with a relative washout calculated at 30%. In magnetic resonance imaging, GN is well-circumscribed masses and encapsulated, it has a homogenously low or intermediate signal intensity in T1-weighted images and heterogeneous slightly high signal intensity on T2-weighted images [15].

In terms of functional exploration, MIBG (131-metaiodobenzylguanidine) scintigraphy produces similar results in GN, ganglioneuroblastoma, and neuroblastomas [5,14]. A PET scan is one of the most helpful modalities to differentiate malignancy and adenoma with 100% sensitivity and 98% specificity [16]. In reality, these radiological features are non-pathognomonic of adrenal GN [15], so the preoperative misdiagnosis rate of adrenal GN based on CT and MRI results was confirmed at 64.7% [6]. Thus, the preoperative differential diagnosis of GN (with ganglioneuroblastoma, neuroblastoma, pheochromocytoma, adenoma, and adrenocortical carcinoma) remains extremely challenging [5,17].

Ultimately, biopsy with histological examination is the current diagnostic gold standard, after the exclusion of pheochromocytoma. In the majority of cases, GN is histologically benign lesions that can be classified into two large groups, mature and maturing types [14]. “Mature type” is composed of mature Schwann cells, ganglion cells, and perineurial cells. “Maturing type” consists of similar cellular populations with miscellaneous maturation degrees, ranging from fully mature cells to neuroblasts. According to immunohistochemical analysis, GN is characterized by reactivity for specific markers such as S-100, vimentin, synaptophysin, and neuron-specific enolase [15]. In our patient, a final pathological examination confirmed the diagnosis.

The treatment of GN is surgical, either laparoscopic or open surgical resection of the tumor [9]. Laparoscopic resection is recommended for tumors less than 6 cm [18]. Postoperatively, there is no need for adjuvant therapy and their prognosis is excellent [8,17].

## Conclusions

Adrenal GNs are rare differentiated tumors that come from neural crest cells. Its location at the adrenal level is unusual. In the vast majority of cases, it is benign tumors often diagnosed by a mass effect, or discovered incidentally during imaging examinations. Preoperative diagnosis is difficult and histology remains the gold standard for diagnostic certainty. The prognosis in these patients is excellent after surgical resection.

The objective of reporting this case is to demonstrate that the presence of an adrenal mass, (especially if it is unilateral), in a neoplastic context, is not always synonymous with metastases and may be related to a benign adrenal tumor (for example, an adrenal ganglioneuroma as is the case of our patient).
